# Supplementary material for: RNA compaction and iterative scanning for small RNA targets by the Hfq chaperone
Source: Nat Commun. 2024 Mar 7;15:2069. doi: 10.1038/s41467-024-46316-6 (PMC10920880; doi:10.1038/s41467-024-46316-6)
Supplement: Supplementary file 1 — Supplementary Information [file 41467_2024_46316_MOESM1_ESM.pdf]

SUPPLEMENTARY INFORMATION

RNA compaction and iterative scanning for small RNA targets by the Hfq chaperone

Ewelina M. Małecka* and Sarah A. Woodson*[1]

T. C. Jenkins Department of Biophysics, Johns Hopkins University, 3400 N. Charles St., Baltimore, MD. 21218. USA

Supplementary Figures S1-S6

Supplementary Tables S1-S4

SUPPLEMENTAL FIGURES

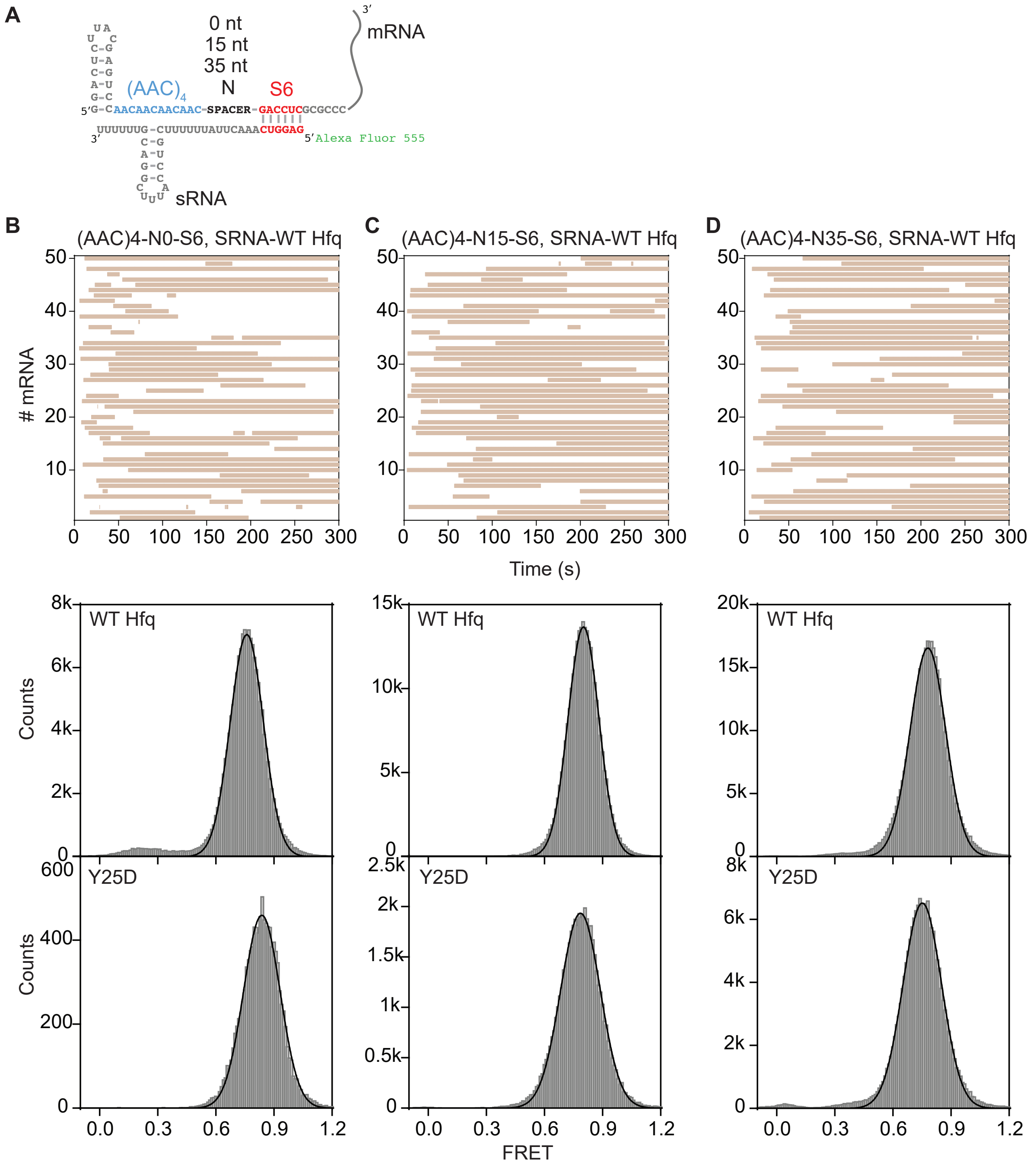

Figure S1. Characterizing the effect of spacer length on sRNA-mRNA annealing efficiency. Related to Figure 1.

(A) Sequences of a designed, minimal sRNA and mRNA pair, containing a 6 bp complementary region (S6, red), a binding site for the distal face of Hfq in the mRNA (blue), and N nt spacer between the Hfq- and sRNA-binding sites. A 3′ extension of the mRNA (gray line) is

complementary to the tether DNA, which is labeled with Cy5. Alexa Fluor 555 is attached to the 5′ end of the sRNA.

(B-D) Summary of smFRET data for sRNA annealing with mRNAs containing spacers (B) N = 0 nt, (C) N = 15 nt, (D) N = 35 nt. (Top panels) Rastergrams depicting sRNA•Hfq binding over time, relative to the moment of adding 5 nM sRNA•Hfq complexes to slides containing immobilized mRNA. 50 randomly selected traces are stacked, and each horizontal bar represents a single binding event. (Middle and bottom panels) sRNA duplexes formed with each mRNA sequence yield the same high FRET state, in the presence of WT Hfq or Hfq:Y25D. Histograms of FRET efficiencies from sRNA•mRNA binding events were fit with a Gaussian function. N_{events} = 154 (N0, WT, *E*~0.76), 16 (N0, Y25D, *E*~0.84), 219 (N15, WT, *E*~0.8), 33 (N15, Y25D, *E*~0.79), 313 (N35, WT, *E*~0.78), 172 (N35, Y25D, *E*~0.75). The small amount of data for Hfq:Y25D is due to poor recruitment of the sRNA-Hfq:Y25D complex to mRNAs. The FRET efficiency of the Hfq:Y25D complex on N0 mRNA may be slightly higher than the FRET efficiency of the other complexes because the mRNA is positioned somewhat differently on Hfq.

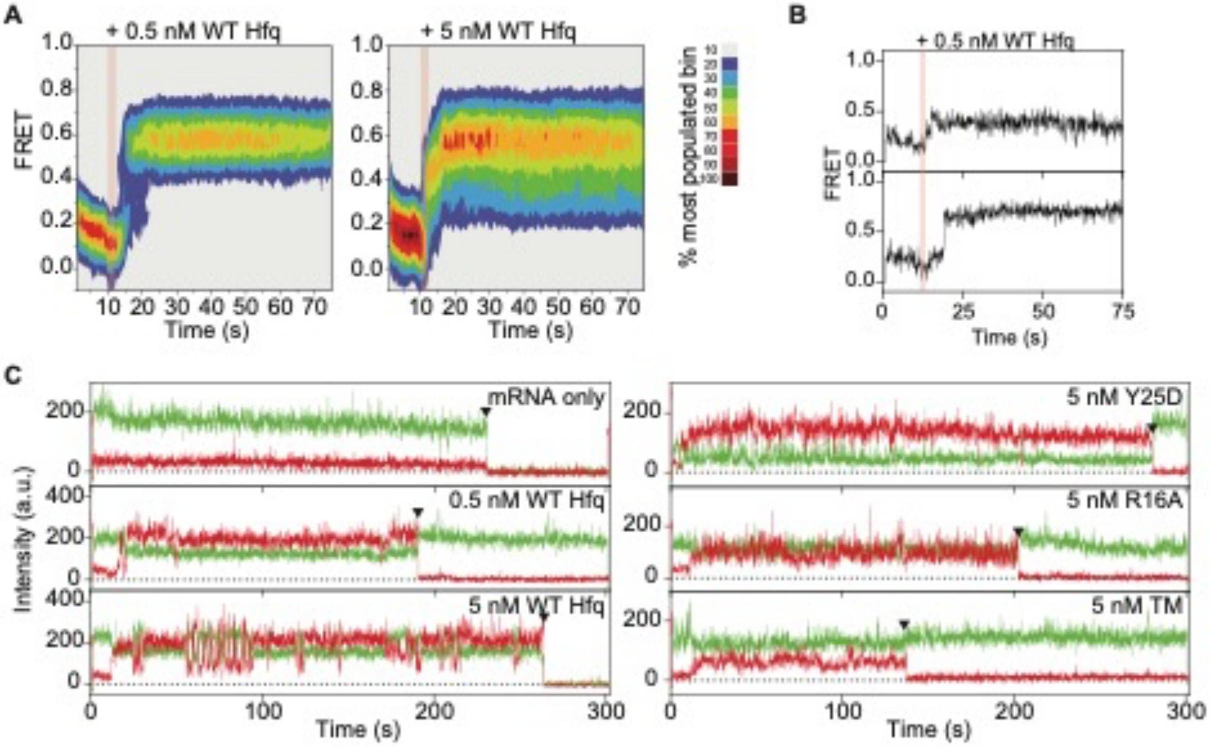

Figure S2. Characterizing Hfq-mediated mRNA compaction. Related to Figure 2.

(A) Two-dimensional histograms of time-resolved FRET trajectories in which 0.5 nM Hfq (left, 145 mRNAs) or 5 nM WT Hfq (right, 134 mRNAs) was injected between the 100th and 130th frame (pink stripe). The population of each pixel was colored relative to the most populated pixel, from blue (10%) to dark red (100%). The mRNA fluctuates between FRET states in 5 nM Hfq (see below), producing a broad distribution of FRET values after 20 s.

(B) FRET trajectories for two representative immobilized mRNAs (AAC)6-Cy5-N35-S6 upon injection of indicated 0.5 nM WT Hfq. The moment of Hfq injection is marked (pink stripe).

(C) Time trajectories for representative immobilized mRNAs (AAC)6-Cy5-N35-S6 indicating donor (green) and acceptor (red) intensity upon the injection of the indicated Hfq variants. Photobleaching events are marked with arrows. TM, rim triple mutant R16A, R17A, R19D.

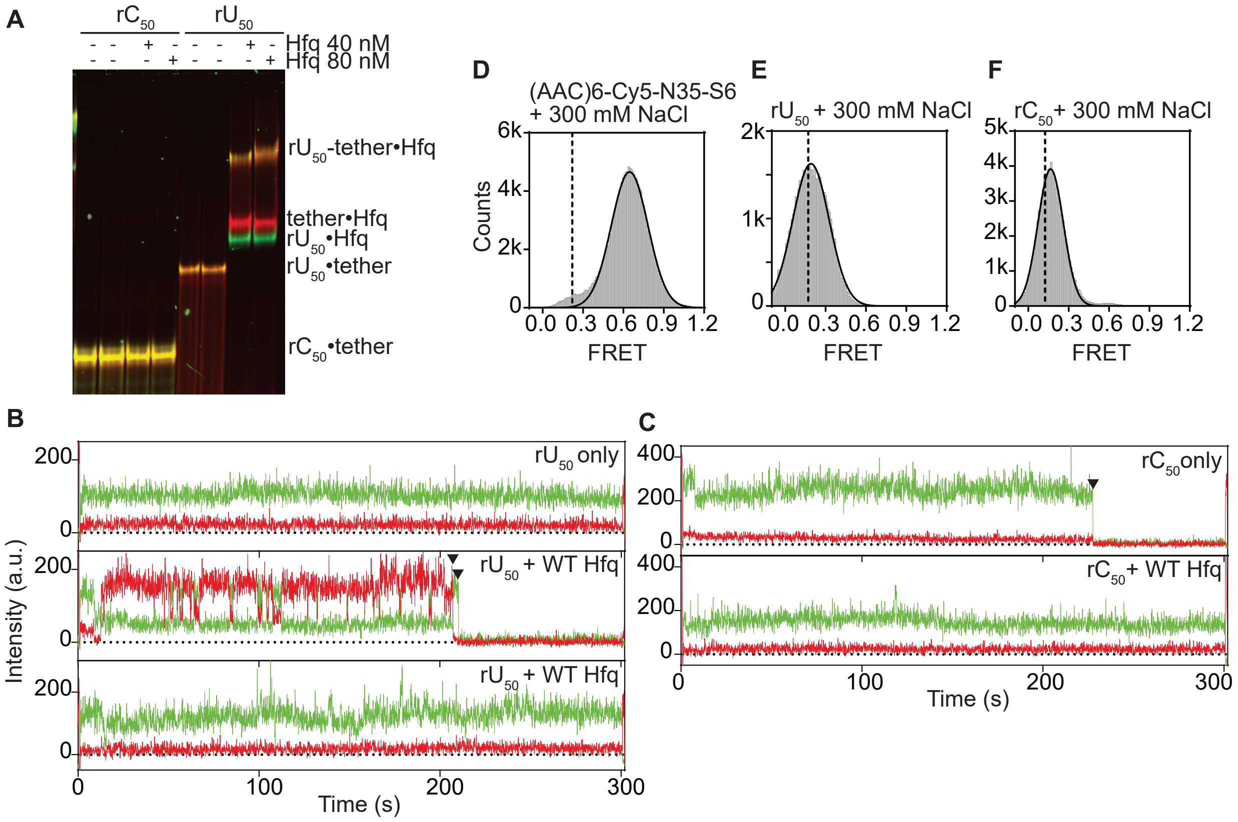

Figure S3. Effect of Hfq on the end-to-end distance of RNA homopolymers. Related to Figure 3.

(A) Binding of Cy3-labeled homopolymers annealed to Cy5-labeled DNA tether to Hfq in a native 8% PAGE mobility shift assay. Red, Cy5 scan; green, Cy3 scan; orange, overlay. The complexes corresponding to each band are marked on the right.

(B, C) Time trajectories for representative immobilized (B) rU_{50} or (C) rC_{50} molecules, indicating donor (green) and acceptor (red) intensity upon the injection of indicated Hfq variants. Photobleaching events are marked with arrows.

(D-F) The distribution of FRET values in high salt buffer (50 mM Tris-HCl pH 7.5, 300 mM NaCl). Dashed line represents the E_{FRET} obtained for these RNAs in TNK buffer. (D) (AAC)6-Cy5-N35-S6 (N = 327 molecules), (E) rU_{50} (N = 93 molecules), (F) rC_{50} (N = 160 molecules).

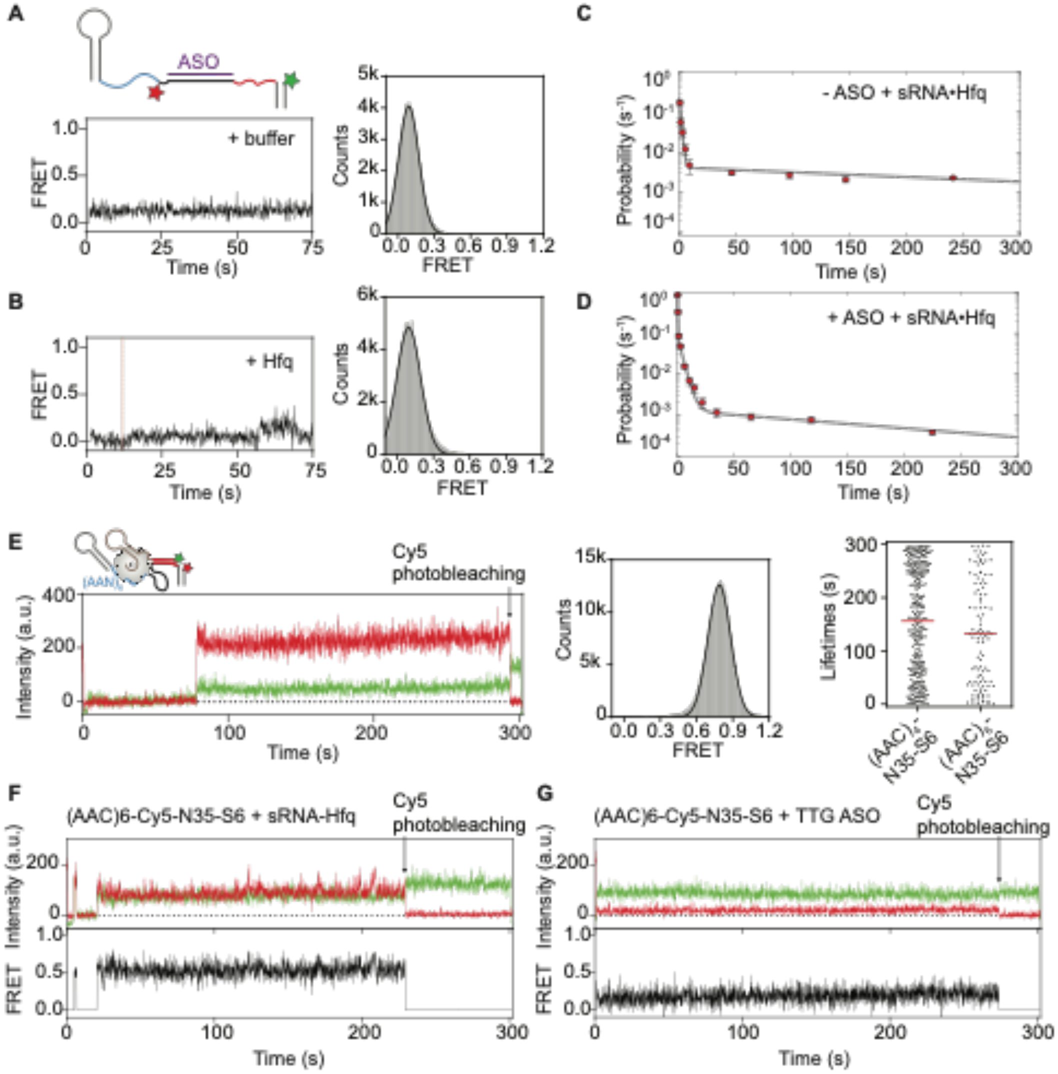

Figure S4. A flexible mRNA spacer is required for sRNA annealing to distant binding sites in the minimal mRNA. Related to Figure 4.

(A, B) Conformation of immobilized mRNA (AAC)6-Cy5-N35-S6 annealed to a 28 nt antisense oligonucleotide (ASO) complementary to the spacer between the Hfq and sRNA binding sites. (A) Before Hfq addition; (B) Over the first minute after injection of 5 nM Hfq. (Left panels) Representative FRET trajectories; the pink stripe indicates the time of Hfq addition. (Right panels) Distribution of FRET values. (A) N = 149 molecules (*E*~0.09), (B) N = 215 molecules (*E*~0.1).

(C, D) Probability density of sRNA-Hfq residence lifetimes on (AAC)6-Cy5-N35-S6 mRNA; (C) –ASO (N = 322 events) or (D) +ASO (N = 752 events). Lines represent maximum likelihood (MLE) fits to equations with two (C, $\tau_1 = 1.3 \pm 0.3$ s, $\tau_2 = 370 \pm 50$ s, a = 0.15 ± 0.05) or three (D, $\tau_1 = 0.26 \pm 0.03$ s, $\tau_2 = 3.9 \pm 0.6$ s, $\tau_3 = 199 \pm 44$ s, $a_1 = 0.49 \pm 0.03$, $a_2 = 0.29 \pm 0.02$) exponential terms. Error bars represent the SD of a binomial distribution.

(E) Alexa Fluor 555-sRNA-Hfq binding to (AAC)6-N35-S6 mRNA tethered to Cy5-labeled DNA. High FRET efficiency reports on sRNA-mRNA base pairing. (Left) Representative fluorescence trajectory. (Center) sRNA duplexes formed with (AAC)6-N35-S6 mRNA yield a high FRET state (N = 270 events). (Right) Lifetimes of sRNA•Hfq bound to mRNAs with 4 or 6 AAC triplets in the Hfq binding site, showing that they have comparable stability. Red lines indicate the mean. The slightly shorter average lifetime of (AAC)6-N35-S6 complexes may be due to the smaller number of observations and is likely not significant. (AAC)4-N35-S6 is the same as N=35 in Fig. 1D.

(F) Representative fluorescence trajectory for sRNA-Hfq binding to (AAC)6-Cy5-N35-S6 mRNA tethered to unlabeled DNA.

(G) Representative fluorescence trajectory for TTG antisense nucleotide (ASO) binding to (AAC)6-Cy5-N35-S6 mRNA tethered to unlabeled DNA.

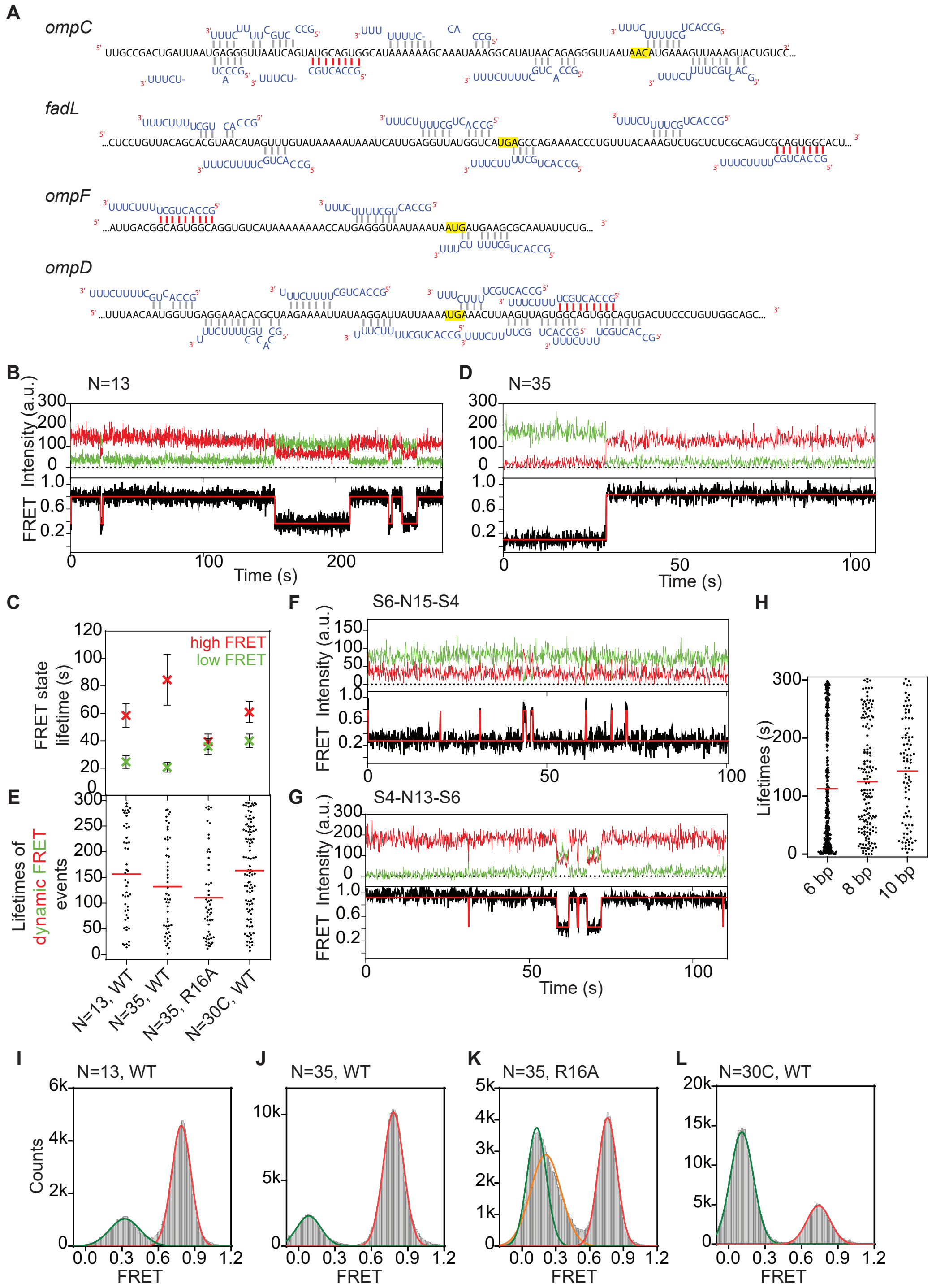

Figure S5. Kinetics of sRNA transfer between sites in single mRNA molecules. Related to Figures 5 and 6.

(A) Cryptic sRNA target sites in natural mRNA regulatory targets. Examples show base complementarity between the RybB sRNA seed sequence (blue) and four target mRNAs (black). The base pairs shown in red were proposed to be responsible for RybB regulation [S1]. Base pairs

shown in gray were predicted by RNAstructure [S2], using 20 nt mRNA fragments with a 10 nt window size. The start codon for each ORF is highlighted in yellow.

(B, D, F, G) Representative trajectories for dynamic binding of Alexa Fluor 555-sRNA-Hfq with tandem mRNAs. (Top) Fluorescence intensities of Alexa Fluor 555 (green) and Cy5 (red). (Bottom) The corresponding FRET efficiency (black) with the FRET states fitted using ebFRET (red line). (B) (AAC)4-S6-N13-S6 mRNA, (D) (AAC)4-S6-N35-S6 mRNA, (F) (AAC)4-S6-N15-S4 mRNA, (G) (AAC)4-S4-N13-S6 mRNA.

(C) Scatter plot representing the average residence times of the sRNA residing at each target site in tandem mRNAs with different spacers or in the presence of the indicated Hfq variants. Red symbols, site far from AAC motif (high FRET), green symbols, site adjacent to AAC motif (low FRET). Error bars represent the SD of a binomial distribution.

(E) Lifetimes of dynamic sRNA binding events on indicated mRNAs in the presence of the indicated Hfq variants. The mean is marked with a red line: N=13 WT, 156 s (N=49 events); N=35 WT, 132 s (N=50 events); N=35 R16A, 110 s (N= 52 events), N=30C WT, 163 s (N=101 events).

(H) Distributions of sRNA-mRNA dwell times for mRNAs with different extent of sRNA-mRNA base pairing. The mean is marked with a red line: 6 bp, 112 s (N=154 events); 8 bp, 125 s (N=158 events); 10 bp, 143 s (N= 91 events). A fraction of binding events persisted until the end of the movie (300 s); (6 bp, 32%, 8 bp, 53%, 10 bp, 62%). mRNA 6 bp corresponds to "N=0" on Fig. 1D.

(I-L) Target site binding. The distribution of FRET values for all sRNA-Hfq binding events on the indicated mRNAs in the presence of the indicated Hfq variants. (H) (AAC)4-S6-N13-S6 mRNA, WT Hfq (N = 127 events; 112 molecules), (I) (AAC)4-S6-N35-S6 mRNA, WT Hfq (N = 226 events; 201 molecules) (J) (AAC)4-S6-N35-S6 mRNA, R16A (N = 245 events; 218 molecules) (K) (AAC)4-S6-N30C-S6 mRNA, WT Hfq (343 events; 323 molecules). The equilibria were obtained from the area under the Gaussian curve for each peak.

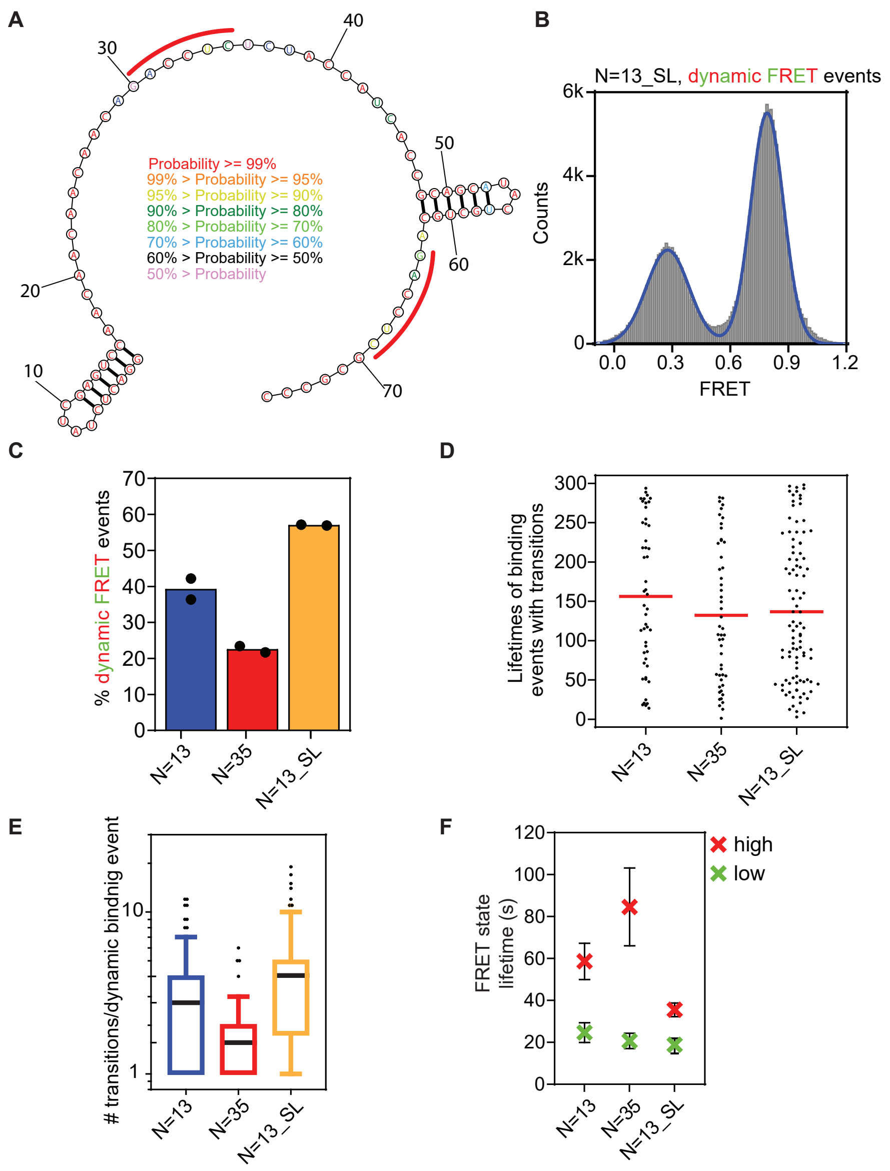

Figure S6. Hfq can bypass RNA secondary structure during target scanning. Related to Figures 5 and 6.

(A) Secondary structure of a tandem (AAN)4-S6-N13-SL-S6 mRNA (N=13_SL), containing a 13 nt single-stranded spacer plus a stem-loop between two target sites. sRNA binding sites are marked with red lines.

(B) Histogram of FRET efficiency of sRNA complexes with N=13_SL mRNA showing shuttling between low FRET and high FRET states (dynamic events).

(C) Percent of all sRNA binding events that are dynamic, for tandem mRNAs containing spacers N = 13, N = 35, and N = 13_SL. Symbols indicate the values of independent trials; bar height indicates the mean.

(D) Dwell times of sRNA-Hfq binding to each mRNA shown, for dynamic binding events that result in transfer between low and high FRET states. The mean is marked with a red line: N=13_SL, 136 s (N=98 events) and Fig. S5E.

(E) The number of transitions between low and high FRET states for each sRNA-Hfq binding event. The whiskers are drawn from the 10th to the 90th percentile; box indicates first to third quartile. Mean values of transition numbers per mRNA (black horizontal line) are: 4 for N = 13_SL (N = 98 events) and see Fig. 5J.

(F) Lifetimes of low FRET and high FRET states for dynamic sRNA-Hfq-mRNA complexes. Red symbols, site far from AAC motif (high FRET), green symbols, site adjacent to AAC motif (low FRET). Error bars represent the SD of a binomial distribution. Data for N=35 and N=13 are the same as in Fig. S5C.

SUPPLEMENTAL TABLES

Table S1. Oligonucleotides used in the study.

Name	Sequence	Source	Identifier
Bio-SA5-Cy5	biotin-CCTGTGTCCTGTGTGTCCTGTCCAAAGTGT GTCGTCC/3Cy5/	IDT.	(Małecka and Woodson, 2021)[S3]
Bio-SA5-Cy3	biotin-CCTGTGTCCTGTGTGTCCTGTCCAAAGTGT GTCGTCC/3Cy3/	IDT	This study
Bio-SA5	biotin-CCTGTGTCCTGTGTGTCCTGTCCAAAGTGT GTCGTCC	IDT	This study
(AAC)6-Cy5-N35-S6_splint (Cy5-6BP-35INS_splintLig2)	GTAAAGTTTAGGTGATGCAGGGGTTGTTGTTGTT	Thermo Fisher	This study
5AmC6-AntiAAN5end-15bp-6BP-35INS	/5AmMC6/TTGTTGTTGTTGTTG	Thermo Fisher	This study
TTG_ASO (5AmC6-AntiComp-11bp-6BP-35INS)	/5AmMC6/GAGGTCTGGTG	Thermo Fisher	This study
Anti-Cy5_6BP-35INS	TGATGGTAGAGTAAAGTTTAGGTGATGC	Thermo Fisher	This study
RNA tether:	biotin-UGGCGACGGCAGCGAGGC-/3Cy5/	IDT	(Niaki et al., 2020)
	/5Cy3/(U)$_{50}$GCCUCGCUGCCGUCGCCA	IDT	(Niaki et al., 2020)
	/5Cy3/(C)$_{50}$GCCUCGCUGCCGUCGCCA	IDT	(Niaki et al., 2020)

Table S2. Primers for transcription templates used in the study.

Name	Sequence	Source	Identifier
(AAC)6-Cy5-N35-S6-5part (5'mRNAyWT+C)	GGACUCUAUCGAGUCCAACAACAACAACAACAACC/intCy5/CCU	IDT	(Małecka and Woodson, 2021)[S3]
(AAC)6-Cy5-N35-S6-3part_F (Cy5-6BP+35INS-3part_F)	TAATACGACTCACTATAGCATCACCTAAACTTTACTCTACCATCACCAGACCTCGCGCCC	Thermo Fisher	This study
(AAC)6-Cy5-N35-S6-3part_R (Cy5-6BP+35INS-3part_R)	CCTGTGTCCTGTGTGTCCTGTCCAAAGTGTGTCGTCCTGGGCGCGAGGTCTGGTGATGG	Thermo Fisher	This study
sRNA_F (sRNA_shuttle_F)	TAATACGACTCACTATAGAGGTCAAACTTATTTTTTCGTCCA	Thermo Fisher	This study
sRNA_R (sRNA_shuttle_R)	AAAAAACGTCCGAAATGGACGAAAAAAATAAGTTTGACC	Thermo Fisher	This study
(AAC)4-N0-S6_F (mRNA_6bp_F)	TAATACGACTCACTATAGGACTCTATCGAGTCCAACAACAACAACGACCTCGCGCCCAGGACGACAC	Thermo Fisher	This study
(AAC)4-N0-S6_R (mRNA_shuttle_R)	CCTGTGTCCTGTGTGTCCTGTCCAAAGTGTGTCGTCCTGGGCGCGAGGT	Thermo Fisher	This study
(AAC)4-N15-S6_F (mRNA_6bp+15INS_F)	TAATACGACTCACTATAGGACTCTATCGAGTCCAACAACAACAACTCTACCATCACCTAAGACCTCG	Thermo Fisher	This study
(AAC)4-N15-S6_R (mRNA_6bp+15INS_R)	CCTGTGTCCTGTGTGTCCTGTCCAAAGTGTGTCGTCCTGGGCGCGAGGTCTTAGGTGATGGTAG	Thermo Fisher	This study
(AAC)4-N35-S6_F (mRNA_6BP-35INS_F)	TAATACGACTCACTATAGGACTCTATCGAGTCCAACAACAACAACTCTACCATCACCTAAACTTTACTCTACCATC	Thermo Fisher	This study
(AAC)4-N35-S6_R (mRNA_6BP-35INS_R)	CCTGTGTCCTGTGTGTCCTGTCCAAAGTGTGTCGTCCTGGGCGCGAGGTCTGGTGATGGTAGAGTAAAGTTTAGG	Thermo Fisher	This study
(AAC)6-N35-S6_F (mRNA_H6_6BP-35INS_F)	TAATACGACTCACTATAGGACTCTATCGAGTCCAACAACAACAACAACAACCCCTGCATCACCTAAACTTTACTCTACCATC	Thermo Fisher	This study
(AAC)6-N35-S6 (mRNA_6BP-35INS_R)	CCTGTGTCCTGTGTGTCCTGTCCAAAGTGTGTCGTCCTGGGCGCGAGGTCTGGTGATGGTAGAGTAAAGTTTAGG	Thermo Fisher	This study
(AAC)4-S6-N13-S6_F (mRNA_shuttle2_H1_6BP_F)	TAATACGACTCACTATAGGACTCTATCGAGTCCAACAACAACAACAGACCTCTCTACCATCACCAGACCTCGCGCCCAGGACGACAC	Thermo Fisher	This study
(AAC)4-S6-N13-S6_R (mRNA_shuttle2_H1A6_noSL_R)	CCTGTGTCCTGTGTGTCCTGTCCAAAGTGTGTCGTCCTGGGCGCGAGG	Thermo Fisher	This study
(AAC)4-S6-N35-S6_F (mRNA_shtl_H1_6BP-35INS_F)	TAATACGACTCACTATAGGACTCTATCGAGTCCAACAACAACAACAGACCTCTCTACCATCACCTAAACTTTACTCTACCATC	Thermo Fisher	This study
(AAC)4-S6-N35-S6_R (mRNA_6BP-35INS_R)	CCTGTGTCCTGTGTGTCCTGTCCAAAGTGTGTCGTCCTGGGCGCGAGGTCTGGTGATGGTAGAGTAAAGTTTAGG	Thermo Fisher	This study
(AAC)4-S6-N15-S4_R (mRNA_shuttle2_H1_6-4BP_F)	TAATACGACTCACTATAGGACTCTATCGAGTCCAACAACAACAACCGACCTCTCTACCATCACCACTCCTCGCGCCCAGGACGACAC	Thermo Fisher	This study
(AAC)4-S4-N13-S6_F (mRNA_shuttle2_H1_4-6BP_F)	TAATACGACTCACTATAGGACTCTATCGAGTCCAACAACAACAACACTCCTCTCTACCATCACCCGACCTCGCGCCCAGGACGACAC	Thermo Fisher	This study

(AAC)4-S6-N30C-S6_F (mRNA_shtl_H1_6BP-30INS-C_F)	TAATACGACTCACTATAGGACTCTATCGAGTCCAACAACAACAACAGACCTCCCCCCCCCCCCCCCCCCCCCCCCCCCCCCGACCTCGCGCCCAGGAC	Thermo Fisher	This study
(AAC)4-S6-N30C-S6_R (mRNA_shtl_H1_6BP-30INS-C_R)	CCTGTGTCCTGTGTGTCCTGTCCAAAGTGTGTCGTCCTGGGCGCGAGGTCGG	Thermo Fisher	This study
(AAC)4-S6-N13-S6_SL_F (mRNA_shtl2_H1_6BP_SL_F)	TAATACGACTCACTATAGGACTCTATCGAGTCCAACAACAACAACAGACCTCTCTACCATCACCGCAGCATACTGCTGCAGACCTCGCGCCCAGGACG	Thermo Fisher	This study
(AAC)4-S6-N13-S6_SL_R (mRNA_shtl2_6bp_R)	CCTGTGTCCTGTGTGTCCTGTCCAAAGTGTGTCGTCCTGGGCGCGAGGTCTG	Thermo Fisher	This study

Table S3. RNAs generated by *in vitro* transcription used in the study. Red – complementary region, blue – $(AAC)_n$, yellow – mRNA extension complementary to DNA tether.

Name	Full RNA sequence
sRNA	/5Alexa Fluor 555/GAGGUCAAACUUAUUUUUUCGUCCAUUUCGGACGUUUUUU
(AAC)4-N0-S6	GGACUCUAUCGAGUCCAACAACAACAACGACCUCGCGCCCAGGACGACACACUUUGGACAGGACACACAGGACACAGG
(AAC)4-N15-S6	GGACUCUAUCGAGUCCAACAACAACAACUCUACCAUCACCUAAGACCUCGCGCCCAGGACGACACACUUUGGACAGGACACACAGGACACAGG
(AAC)4-N35-S6	GGACUCUAUCGAGUCCAACAACAACAACUCUACCAUCACCUAAACUUUACUCUACCAUCACCAGACCUCGCGCCCAGGACGACACACUUUGGACAGGACACACAGGACACAGG
(AAC)6-Cy5-N35-S6	GGACUCUAUCGAGUCCAACAACAACAACAACAACC/intCy5/CCUGCAUCACCUAAACUUUACUCUACCAUCACCAGACCUCGCGCCCAGGACGACACACUUUGGACAGGACACACAGGACACAGG
(AAC)6-N35-S6	GGACUCUAUCGAGUCCAACAACAACAACAACAACCCCUGCAUCACCUAAACUUUACUCUACCAUCACCAGACCUCGCGCCCAGGACGACACACUUUGGACAGGACACACAGGACACAGG
(AAC)4-S6-N13-S6	GGACUCUAUCGAGUCCAACAACAACAACAGACCUCUCUACCAUCACCAGACCUCGCGCCCAGGACGACACACUUUGGACAGGACACACAGGACACAGG
(AAC)4-S6-N35-S6	GGACUCUAUCGAGUCCAACAACAACAACAGACCUCUCUACCAUCACCUAAACUUUACUCUACCAUCACCAGACCUCGCGCCCAGGACGACACACUUUGGACAGGACACACAGGACACAGG
(AAC)4-S6-N15-S4	GGACUCUAUCGAGUCCAACAACAACAACCGACCUCUCUACCAUCACCACUCCUCGCGCCCAGGACGACACACUUUGGACAGGACACACAGGACACAGG
(AAC)4-S4-N13-S6	GGACUCUAUCGAGUCCAACAACAACAACACUCCUCUCUACCAUCACCCGACCUCGCGCCCAGGACGACACACUUUGGACAGGACACACAGGACACAGG
(AAC)4-S6-N30C-S6	GGACUCUAUCGAGUCCAACAACAACAACAGACCUCCCCCCCCCCCCCCCCCCCCCCCCCCCCCCGACCUCGCGCCCAGGACGACACACUUUGGACAGGACACACAGGACACAGG
AAC)4-S6-N13-S6_SL	GGACUCUAUCGAGUCCAACAACAACAACAGACCUCUCUACCAUCACCGCAGCAUACUGCUGCAGACCUCGCGCCCAGGACGACACACUUUGGACAGGACACACAGGACACAGG

Table S4. Summary of the effect of Hfq rim mutation on sRNA-mRNA regulation in *E. coli*.[a]

sRNA	mRNA	effect of Hfq: R16A	Δnt Hfq to sRNA sites
DsrA, ArcZ	*rpoS*	deleterious	80
McaS	*flhD*	deleterious	47
ArcZ	*flhD*	moderate	20 (site B)<br>11 (site A)
ChiX	*chiP*	moderate	2 (ChiX)<br>10 (chiP)
RyhB	*sodB*	no effect	8
RyhB	*sdhC*	no effect	3
RybB	*sdhC*	?	0
Spot42	*sdhC*	?	13

[a]Data from Zhang et al [S5].

SUPPLEMENTAL REFERENCES

S1 Papenfort, K., Bouvier, M., Mika, F., Sharma, C. M. & Vogel, J. Evidence for an autonomous 5’ target recognition domain in an Hfq-associated small RNA. *Proc Natl Acad Sci U S A* **107**, 20435–20440 (2010).

S2 Reuter, J. S. & Mathews, D. H. RNAstructure: Software for RNA secondary structure prediction and analysis. *BMC Bioinformatics* **11**, (2010).

S3 Małecka, E. M. & Woodson, S. A. Stepwise sRNA targeting of structured bacterial mRNAs leads to abortive annealing. *Mol Cell* **81**, 1988-1999.e4 (2021).

S4 Niaki, A. G. *et al.* Loss of Dynamic RNA Interaction and Aberrant Phase Separation Induced by Two Distinct Types of ALS/FTD-Linked FUS Mutations. *Mol Cell* **77**, 82-94.e4 (2020).

S5 Zhang, A., Schu, D. J., Tjaden, B. C., Storz, G. & Gottesman, S. Mutations in interaction surfaces differentially impact E. coli Hfq association with small RNAs and their mRNA targets. *J Mol Biol* **425**, 3678–3697 (2013)
