# Supplementary material for: RNA compaction and iterative scanning for small RNA targets by the Hfq chaperone
Source: Nat Commun. 2024 Mar 7;15:2069. doi: 10.1038/s41467-024-46316-6 (PMC10920880; doi:10.1038/s41467-024-46316-6)
Supplement: Supplementary file 3 — Inventory of Supplementary Information [file 41467_2024_46316_MOESM3_ESM.pdf]

LIST -- SUPPLEMENTARY INFORMATION

RNA compaction and iterative scanning for small RNA targets by the Hfq chaperone

Ewelina M. Małecka and Sarah A. Woodson

T. C. Jenkins Department of Biophysics, Johns Hopkins University, 3400 N. Charles St., Baltimore, MD. 21218. USA

Supplementary Figures S1-S6

Figure S1. Characterizing the effect of spacer length on sRNA-mRNA annealing efficiency. Related to Figure 1.

Figure S2. Characterizing Hfq-mediated mRNA compaction. Related to Figure 2.

Figure S3. Effect of Hfq on the end-to-end distance of RNA homopolymers. Related to Figure 3.

Figure S4. A flexible mRNA spacer is required for sRNA annealing to distant binding sites in the minimal mRNA. Related to Figure 4.

Figure S5. Kinetics of sRNA transfer between sites in single mRNA molecules. Related to Figures 5 and 6.

Figure S6. Hfq can bypass RNA secondary structure during target scanning. Related to Figures 5 and 6.

Supplementary Tables S1-S4

Table S1. Oligonucleotides used in the study.

Table S2. Primers for transcription templates used in the study.

Table S3. RNAs generated by *in vitro* transcription used in the study.

Table S4. Summary of the effect of Hfq rim mutation on sRNA-mRNA regulation in *E. coli*.

Supplementary References.
