# Supplementary material for: RNA compaction and iterative scanning for small RNA targets by the Hfq chaperone
Source: Nat Commun. 2024 Mar 7;15:2069. doi: 10.1038/s41467-024-46316-6 (PMC10920880; doi:10.1038/s41467-024-46316-6)
Supplement: Supplementary file 4 — Reporting Summary [file 41467_2024_46316_MOESM4_ESM.pdf]

nature portfolio

Corresponding author(s): Malecka and Woodson

Last updated by author(s): 2/1/2024

Reporting Summary

Nature Portfolio wishes to improve the reproducibility of the work that we publish. This form provides structure for consistency and transparency in reporting. For further information on Nature Portfolio policies, see our Editorial Policies and the Editorial Policy Checklist.

Statistics

For all statistical analyses, confirm that the following items are present in the figure legend, table legend, main text, or Methods section.

n/a	Confirmed	
☐	☒	The exact sample size (*n*) for each experimental group/condition, given as a discrete number and unit of measurement
☐	☒	A statement on whether measurements were taken from distinct samples or whether the same sample was measured repeatedly
☒	☐	The statistical test(s) used AND whether they are one- or two-sided<br>*Only common tests should be described solely by name; describe more complex techniques in the Methods section.*
☒	☐	A description of all covariates tested
☒	☐	A description of any assumptions or corrections, such as tests of normality and adjustment for multiple comparisons
☐	☒	A full description of the statistical parameters including central tendency (e.g. means) or other basic estimates (e.g. regression coefficient) AND variation (e.g. standard deviation) or associated estimates of uncertainty (e.g. confidence intervals)
☒	☐	For null hypothesis testing, the test statistic (e.g. *F*, *t*, *r*) with confidence intervals, effect sizes, degrees of freedom and *P* value noted<br>*Give P values as exact values whenever suitable.*
☐	☒	For Bayesian analysis, information on the choice of priors and Markov chain Monte Carlo settings
☒	☐	For hierarchical and complex designs, identification of the appropriate level for tests and full reporting of outcomes
☒	☐	Estimates of effect sizes (e.g. Cohen's *d*, Pearson's *r*), indicating how they were calculated

Our web collection on statistics for biologists contains articles on many of the points above.

Software and code

Policy information about availability of computer code

Data collection	Custom smCamera code for movie acquisition is described in Ref. 56 and available from Dr. Taekjip Ha upon request.
Data analysis	Custom IDL code for image analysis is described in Ref. 56 and available from Dr. Taekjip Ha upon request. FRET states were analyzed with ebFRET, http://ebfret.github.io. Binding dwell times were analyzed with Imscroll: https://github.com/gelles-brandeis/CoSMoS_Analysis/tree/master/imscroll.

For manuscripts utilizing custom algorithms or software that are central to the research but not yet described in published literature, software must be made available to editors and reviewers. We strongly encourage code deposition in a community repository (e.g. GitHub). See the Nature Portfolio guidelines for submitting code & software for further information.

Data

Policy information about availability of data

All manuscripts must include a data availability statement. This statement should provide the following information, where applicable:

- Accession codes, unique identifiers, or web links for publicly available datasets
- A description of any restrictions on data availability
- For clinical datasets or third party data, please ensure that the statement adheres to our policy

The data and any additional information required to reanalyze the data reported in this paper are available from Johns Hopkins University Data Services repository, doi.org/10.7281/T1/YPUEQT

Research involving human participants, their data, or biological material

Policy information about studies with human participants or human data. See also policy information about sex, gender (identity/presentation), and sexual orientation and race, ethnicity and racism.

Reporting on sex and gender	Not applicable; no human subjects
Reporting on race, ethnicity, or other socially relevant groupings	No human subjects
Population characteristics	No human subjects
Recruitment	No human subjects
Ethics oversight	No human subjects

Note that full information on the approval of the study protocol must also be provided in the manuscript.

Field-specific reporting

Please select the one below that is the best fit for your research. If you are not sure, read the appropriate sections before making your selection.

☒ Life sciences ☐ Behavioural & social sciences ☐ Ecological, evolutionary & environmental sciences

For a reference copy of the document with all sections, see nature.com/documents/nr-reporting-summary-flat.pdf

Life sciences study design

All studies must disclose on these points even when the disclosure is negative.

Sample size	The numbers of single molecules analyzed for each experiment are provided in the figure legends. The sample size was not pre-determined; all technically valid data were analyzed and presented. Data collection was continued until physical parameters could be obtained with the precision given in the paper.
Data exclusions	All data were included, Data were only excluded for technical reasons (inactive fluorophores, etc); these criteria are described in the methods.
Replication	Each experiment was performed 2-4 times (usually 3); these are completely independent trials. All replicates are presented and included in the analyses.
Randomization	Not applicable. Observations in response to independent variables are reported; there is no natural population from which a subset of samples must be selected. In a few figures, a subset of the data were randomly selected for visualization, but all data were included in the analyses.
Blinding	Not applicable. This is a physical investigation of biochemical pathways, and hypotheses about these pathways.

Reporting for specific materials, systems and methods

We require information from authors about some types of materials, experimental systems and methods used in many studies. Here, indicate whether each material, system or method listed is relevant to your study. If you are not sure if a list item applies to your research, read the appropriate section before selecting a response.

Materials & experimental systems

n/a	Involved in the study	
☒	☐	Antibodies
☒	☐	Eukaryotic cell lines
☒	☐	Palaeontology and archaeology
☒	☐	Animals and other organisms
☒	☐	Clinical data
☒	☐	Dual use research of concern
☒	☐	Plants

Methods

n/a	Involved in the study	
☒	☐	ChIP-seq
☒	☐	Flow cytometry
☒	☐	MRI-based neuroimaging

Plants

Seed stocks	Not applicable (no plants)
Novel plant genotypes	N/A
Authentication	N/A
